# *Fusarium* Wilt Invasion Results in a Strong Impact on Strawberry Microbiomes

**DOI:** 10.3390/plants12244153

**Published:** 2023-12-13

**Authors:** Hongjun Yang, Xu Zhang, Xiaohong Qiu, Jiajia Chen, Yuanhua Wang, Geng Zhang, Sizhen Jia, Xiangqi Shen, Wenwu Ye, Zhiming Yan

**Affiliations:** 1 College of Agronomy and Horticulture, Jiangsu Vocational College of Agriculture and Forestry, Zhenjiang 212400, China; hjyang@jsafc.edu.cn (H.Y.); hanyu30082@126.com (X.Q.); wangyuanhua@jsafc.edu.cn (Y.W.); gengzhang@jsafc.edu.cn (G.Z.); jiasizhen@jsafc.edu.cn (S.J.); xiangqishen1355@163.com (X.S.); 2 Jiangsu Engineering and Technology Center for Modern Horticulture, Zhenjiang 212400, China; 3 College of Landscape Architecture, Jiangsu Vocational College of Agriculture and Forestry, Zhenjiang 212400, China; jiajiachen@jsafc.edu.cn; 4 Key Laboratory of Plant Immunity, Department of Plant Pathology, Nanjing Agricultural University, Nanjing 210095, China; yeww@njau.edu.cn

**Keywords:** microbiome, strawberry *Fusarium* wilt disease, fungal community, bacterial community, plant pathogen

## Abstract

Plant-endophytic microbes affect plant growth, development, nutrition, and resistance to pathogens. However, how endophytic microbial communities change in different strawberry plant compartments after *Fusarium* pathogen infection has remained elusive. In this study, 16S and internal transcribed spacer rRNA amplicon sequencing were used to systematically investigate changes in the bacterial and fungal diversity and composition in the endophytic compartments (roots, stems, and leaves) of healthy strawberries and strawberries with *Fusarium* wilt, respectively. The analysis of the diversity, structure, and composition of the bacterial and fungal communities revealed a strong effect of pathogen invasion on the endophytic communities. The bacterial and fungal community diversity was lower in the *Fusarium*-infected endophytic compartments than in the healthy samples. The relative abundance of certain bacterial and fungal genera also changed after *Fusarium* wilt infection. The relative abundance of the beneficial bacterial genera *Bacillus*, *Bradyrhizobium*, *Methylophilus*, *Sphingobium*, *Lactobacillus*, and *Streptomyces*, as well as fungal genera *Acremonium*, *Penicillium*, *Talaromyces*, and *Trichoderma*, were higher in the healthy samples than in the *Fusarium* wilt samples. The relative abundance of *Fusarium* in the infected samples was significantly higher than that in the healthy samples, consistent with the field observations and culture isolation results for strawberry wilt. Our findings provide a theoretical basis for the isolation, identification, and control of strawberry wilt disease.

## 1. Introduction

Endophytic microbes colonize the plant endophytic compartment and considerably influence plants’ growth, development, nutrition, and resistance to pathogens [1,2,3,4,5]. In plants, microbial diversity varies across different niches, with the endophytic microbiome of plant roots being primarily absorbed from the soil and transported to the stems and leaves through extracellular vesicles in the xylem vessels [6,7]. Unlike the process of the endophytic colonization of roots, stems, and leaves, that of the formation of rhizosphere microbial communities appears to be stable and controllable [8,9]. The microbes that typically exist in root and stem tissues are either candidate symbionts or potential pathogens [10]. Therefore, understanding the composition of endophytic microbial communities in plant roots, stems, and leaves is essential for the development and application of agricultural biological fertilizers and the regulation of plant diseases [9,11,12].

Strawberries (*Fragaria* × *ananassa*) are a widely cultivated fruit with high nutritional and economic value [13,14]. Because of the continuous expansion of strawberry plantation areas and the use of long-term continuous cropping, strawberries have become susceptible to several diseases, such as *Fusarium* wilt [15,16]. *Fusarium* wilt is caused by a soil-borne pathogen called *Fusarium oxysporum*, which primarily infects plants such as strawberry, cucumber, tomato, banana, and celery; has a wide range of hosts; and results in major crop losses in fruits and vegetables worldwide [17,18,19,20,21]. Strawberry wilt primarily occurs during the seedling, flowering, and harvest stages of strawberries, with seedlings and soil carriers being the main reasons for recurrence [22,23]. This disease typically infects the roots, stems, leaves, fruit stems, and petioles, and results in delayed plant development, wilt, crown discoloration, and ultimately, plant death [24,25,26,27].

High-throughput sequencing has been used to elucidate the structure of microbial communities and estimate the influence of pathogen infection on these communities. Studies have revealed significant differences in the bacterial and fungal communities in rhizosphere soil surrounding anthracnose-infected strawberries, powdery-mildew-infected strawberries, and healthy strawberry plants [28,29]. They have also indicated significant differences in the bacterial and fungal communities in the rhizosphere soil surrounding healthy bananas and banana plants with *Fusarium* wilt [30]. Another study has revealed significant differences in the bacterial community diversity and composition between healthy tobacco plants and tobacco with bacterial root and stem wilt [10]. However, no study has fully elucidated the changes that occur in the bacterial and fungal microbial communities in the endophytic compartments (roots, stems, and leaves) of strawberry plants during *Fusarium* wilt. Therefore, in the present study, we used 16S and internal transcribed spacer (ITS) rRNA amplicon sequencing to evaluate the changes that occur in the bacterial and fungal communities in the roots, stems, and leaves of healthy and *Fusarium* wilt-infected strawberry plants, respectively. We also evaluated the relative importance of these bacterial and fungal communities in the invasion process of *Fusarium* wilt.

## 2. Results

### 2.1. Microbial Community Diversity and Structure of Healthy and Infected Samples

To compare the endophytic microbial communities in healthy and wilt-infected strawberry plants, we analyzed the bacterial and fungal communities present in the roots, stems, and leaves of such plants by sequencing the V5–V7 region of the 16S rRNA gene and the ITS1 region of the ITS, respectively. A total of 3,103,035 16S sequences and 3,804,986 ITS sequences obtained from 60 samples were analyzed. After chimeric and organellar sequences were excluded, the sequences were grouped into 1705 bacterial OTUs and 421 fungal OTUs, which were clustered at an identity threshold of 97%.

**Figure 1:**
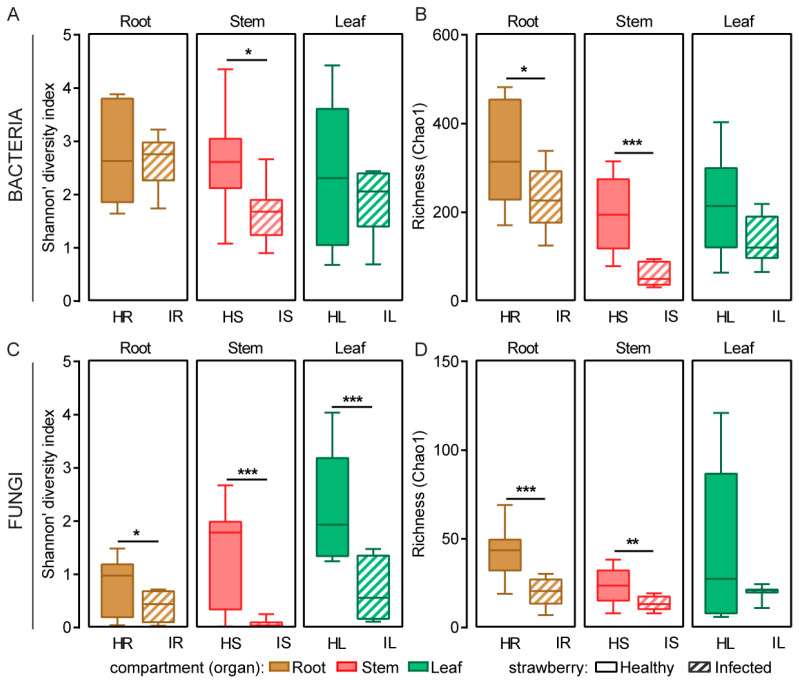
Alpha diversity of bacterial (**A**,**B**) and fungal (**C**,**D**) communities in the roots, stems, and leaves of healthy strawberry plants and strawberries with *Fusarium* wilt. Diversity estimated using Shannon’s index (**A**,**C**) and Chao1 index (**B**,**D**). Statistical analysis conducted using Student’s *t* test (* *p* < 0.05, ** *p* < 0.01, and *** *p* < 0.001). HR: root samples of healthy strawberry plants; IR: root samples of infected strawberry plants; HS: stem samples of healthy strawberry plants; IS: stem samples of infected strawberry plants; HL: leaf samples of healthy strawberry plants; IL: leaf samples of infected strawberry plants.

The alpha diversity indices (Shannon’s and Simpson’s indices) and community richness indices (Chao1, Ace, and Sobs indices) of the bacteria and fungi in the healthy and wilt-infected strawberry roots, stems, and leaves are summarized in Table S1. In the infected root, stem, and leaf samples, the Shannon index and community richness index of the bacteria and fungi exhibited a downward trend compared with those in the healthy root, stem, and leaf samples. The community richness index of the bacteria in the healthy root samples was significantly higher than that in the infected samples (Student’s *t* test, *p* < 0.05), and the Shannon index and community richness index of the bacteria in the healthy stem samples were significantly higher than those in the infected samples (Shannon’s index: *p* < 0.05, Student’s *t* test; Chao1 index: *p* < 0.001, Student’s *t* test; Figure 1A,B; Table S1). The Shannon index of the fungi in the healthy root, stem, and leaf samples was significantly higher than that in the infected samples (roots: *p* < 0.05, Student’s *t* test; stems: *p* < 0.001, Student’s *t* test; leaves: *p* < 0.001, Student’s *t* test), and the community richness index in the healthy root and stem samples was significantly higher than that in the infected samples (roots: *p* < 0.001, Student’s *t* test; stems: *p* < 0.01, Student’s *t* test; Figure 1C,D; Table S1). These results indicate that the number of endophytic bacterial and fungal species in the healthy plants was higher than that in the infected plants.

**Figure 2:**
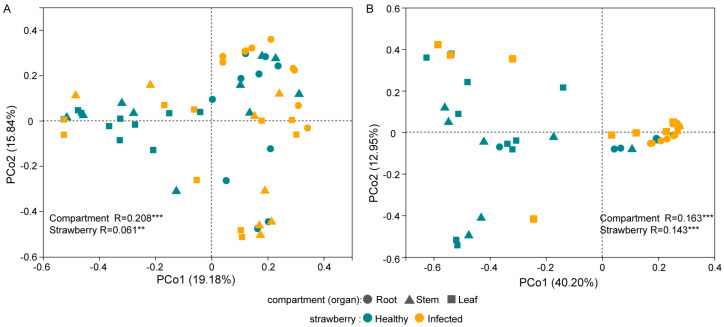
Principal coordinate analysis (PCoA) of bacterial (**A**) and fungal (**B**) communities in healthy strawberry plants and strawberries with *Fusarium* wilt, with Bray–Curtis dissimilarities. Analysis of similarities (ANOSIM) conducted to test for differences in community composition resulting from compartment and health status. *R* values are presented and labeled with asterisks: ** *p* < 0.01 and *** *p* < 0.001.

A principal coordinate analysis combined with an analysis of similarities revealed that compartment and *Fusarium* infection affected the composition of the bacterial and fungal communities (Figure 2A,B). The bacterial community structure in the leaves (*R* = 0.165, *p* < 0.05) of the healthy and infected strawberries underwent the most significant change, whereas the fungal community structure in the stems (*R* = 0.296, *p* < 0.05) of the healthy and infected strawberries underwent the most significant change (Figure S1A,B).

### 2.2. Differences in Bacterial and Fungal Taxa in Healthy and Infected Samples

In the healthy and infected samples, the bacterial OTUs were classified into 36 phyla, 103 classes, 237 orders, 397 families, and 765 genera, and the fungal OTUs were classified into 4 phyla, 14 classes, 28 orders, 48 families, and 63 genera. Because 79.8% and 93.5% of the OTUs in the 16S and ITS data sets were identified as phylum Proteobacteria and phylum Ascomycota, respectively, these groups were further split into classes.

**Figure 3:**
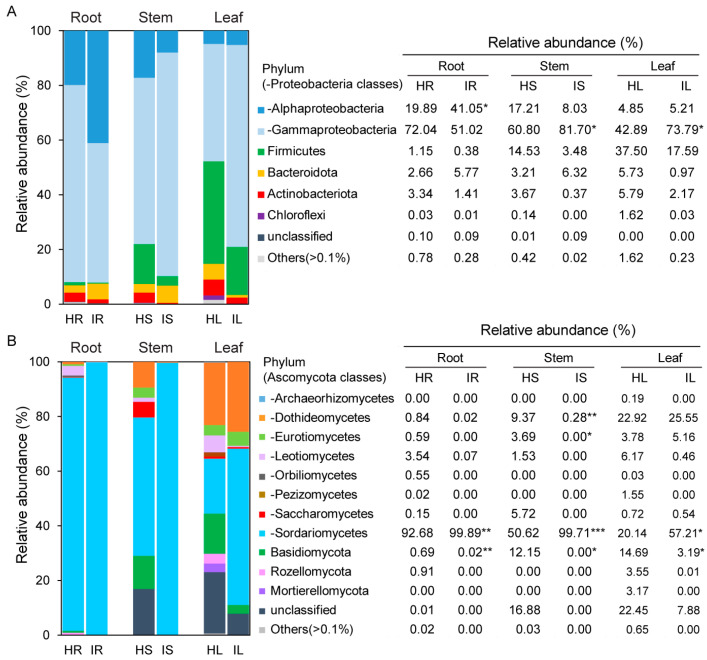
Relative abundance of most abundant (>0.1%) bacterial phyla and Proteobacteria classes (−) (**A**) and fungal phyla and Ascomycetes classes (−) (**B**) in each compartment between healthy strawberries and strawberries with *Fusarium* wilt. Student’s *t* test used to identify significant differences between healthy strawberries and strawberries with *Fusarium* wilt (* *p* < 0.05, ** *p* < 0.01, and *** *p* < 0.001). HR: root samples of healthy strawberry plants; IR: root samples of infected strawberry plants; HS: stem samples of healthy strawberry plants; IS: stem samples of infected strawberry plants; HL: leaf samples of healthy strawberry plants; IL: leaf samples of infected strawberry plants.

Six dominant bacterial phyla and Proteobacteria classes, with a relative abundance of ≥0.1%, were detected (Figure 3A). Among the Proteobacteria classes, Gammaproteobacteria and Alphaproteobacteria were the most dominant, with relative abundances of 63.7% and 16.0%, respectively. Alphaproteobacteria were more abundant in the infected roots than in the healthy roots, and Gammaproteobacteria were more abundant in the infected stems and leaves than in the healthy stems and leaves (*p* < 0.05, Student’s *t* test; Figure 3A). A total of 11 dominant fungal phyla and Ascomycota classes, with a relative abundance of ≥0.1%, were detected (Figure 3B). Among the Ascomycota classes, Sordariomycetes and Dothideomycetes were the most dominant, with relative abundances of 70.0% and 9.8%, respectively. Sordariomycetes were more abundant in the infected roots, stems, and leaves than in the healthy roots, stems, and leaves (roots: *p* < 0.01, Student’s *t* test; stems: *p* < 0.001, Student’s *t* test; leaves: *p* < 0.05, Student’s *t* test), and Basidiomycota were less abundant in the infected roots, stems, and leaves than in the healthy roots, stems, and leaves (roots: *p* < 0.01, Student’s *t* test; stems and leaves: *p* < 0.05, Student’s *t* test; Figure 3B).

**Figure 4:**
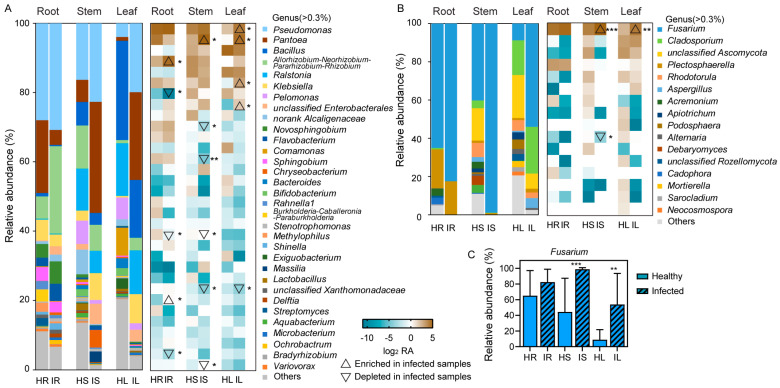
Relative abundance of dominant bacterial (**A**) and fungal (**B**) genera in roots, stems, and leaves of healthy strawberries and strawberries with *Fusarium* wilt (10 replicates). Figure depicts bacterial and fungal genera with relative abundance of >0.3%. Cell colors represent log_2_ fold change in relative abundance compared with control treatment, with brown indicating increasing trend and cyan indicating decreasing trend. (**C**) Relative abundance of the fungal genera *Fusarium.* Student’s *t* test revealed significant differences in relative abundance of bacterial (**A**) and fungal (**B**) genus and *Fusarium* (**C**) (* *p* < 0.05, ** *p* < 0.01, and *** *p* < 0.001) between healthy strawberries and strawberries with *Fusarium* wilt (*n* = 10). HR: root samples of healthy strawberry plants; IR: root samples of infected strawberry plants; HS: stem samples of healthy strawberry plants; IS: stem samples of infected strawberry plants; HL: leaf samples of healthy strawberry plants; IL: leaf samples of infected strawberry plants.

At the genus level, 32 dominant bacterial groups, with a relative abundance of ≥0.3%, were detected. *Allorhizobium*, *Neorhizobium*, *Pararhizobium*, *Rhizobium*, and *Delftia* were more abundant in the infected roots than in the healthy roots, whereas *Pelomonas*, *Methylophilus*, and *Bradyrhizobium* were more abundant in the healthy roots than in the infected roots. *Pantoea* were more abundant in the infected stems than in the healthy stems, whereas *Novosphingobium*, *Sphingobium*, *Methylophilus*, unclassified Xanthomonadaceae, and *Variovorax* were more abundant in the healthy stems than in the infected stems. *Pseudomonas*, *Pantoea*, *Klebsiella*, and unclassified Enterobacterales were more abundant in the infected leaves than in the healthy leaves, whereas unclassified Xanthomonadaceae were more abundant in the healthy leaves than in the infected leaves (Figure 4A). A total of 16 dominant fungal genera, with a relative abundance of ≥0.3%, were identified. *Fusarium* were more abundant in the infected roots, stems, and leaves than in the healthy roots, stems, and leaves (stems: *p* < 0.001, Student’s *t* test; leaves: *p* < 0.01, Student’s *t* test), whereas *Alternaria* were more abundant in the healthy stems than in the infected stems (Figure 4B,C).

### 2.3. Potential Bacterial Metabolic Function and Fungal Functional Guilds of Healthy and Infected Samples

**Figure 5:**
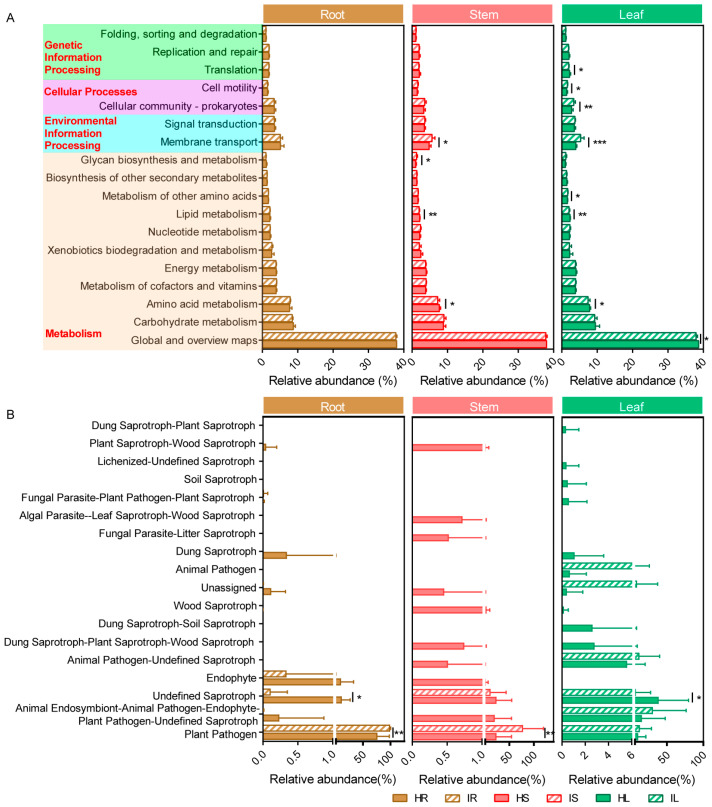
Relative abundance of bacterial (**A**) and fungal (**B**) predicted functional groups (guilds) in healthy and infected roots, stems, and leaves inferred using PICRUSt2 and FUNGuild, respectively. Student’s *t* test revealed significant differences in relative abundance of bacterial metabolic function and fungal functional guilds (* *p* < 0.05, ** *p* < 0.01, and *** *p* < 0.001) between healthy strawberries and strawberries with *Fusarium* wilt (*n* = 10). HR: root samples of healthy strawberry plants; IR: root samples of infected strawberry plants; HS: stem samples of healthy strawberry plants; IS: stem samples of infected strawberry plants; HL: leaf samples of healthy strawberry plants; IL: leaf samples of infected strawberry plants.

A Kyoto Encyclopedia of Genes and Genomes pathway analysis was used to predict the potential functional profiles of the bacterial communities in healthy and infected strawberry samples in PICRUSt. The results indicate that most of the predicted protein sequences in the strawberry samples were clustered into metabolism (74.35%), environmental information processing (8.59%), cellular processes (5.85%), and genetic information processing (5.06%). A total of eleven, three, and two pathways were identified for metabolism, genetic information processing, and environmental information processing and cellular processes, respectively (Figure 5A). The relative abundance of the sequences related to amino acid metabolism and lipid metabolism in the IS and IL was significantly lower in the IS and IL than in the HS and HL, indicating that wilt decreased the degradation of these complex compounds. By contrast, the relative abundance of sequences related to membrane transport was higher in the IS and IL than in the HS and HL. In addition, the relative abundance of the metabolism of other amino acids, cellular community prokaryotes, and cell motility sequences was higher in the IL than in the HL, indicating that wilt increased the degradation of these complex compounds (Figure 5A).

OTUs assigned to a guild with a confidence ranking of “highly probable” or “probable” were retained in the analysis, whereas those with a confidence ranking of “possible” were regarded as unclassified [9]. After annotating the relative abundance of 18 fungal functional guilds, we discovered that the relative abundance of several functional guilds differed between the healthy and wilt-infected samples (Figure 5B). The relative abundance of plant pathogens in the infected root, stem, and leaf samples was higher than that in the healthy root, stem, and leaf samples (roots and stems: *p* < 0.01, Student’s *t* test). The OTU confidence level of *Fusarium* was “possible”; the relative abundance of plant pathogens did not include *Fusarium*. However, the relative abundance of undefined saprotrophs in the infected root, stem, and leaf samples was lower than that in the healthy root, stem, and leaf samples (roots and leaves: *p* < 0.05, Student’s *t* test). In addition, the relative abundance of endophytes in the infected root and stem samples was lower than that in the healthy root and stem samples (Figure 5B).

### 2.4. Differences in Fungal Isolation Taxa between Healthy and Infected Samples

**Figure 6:**
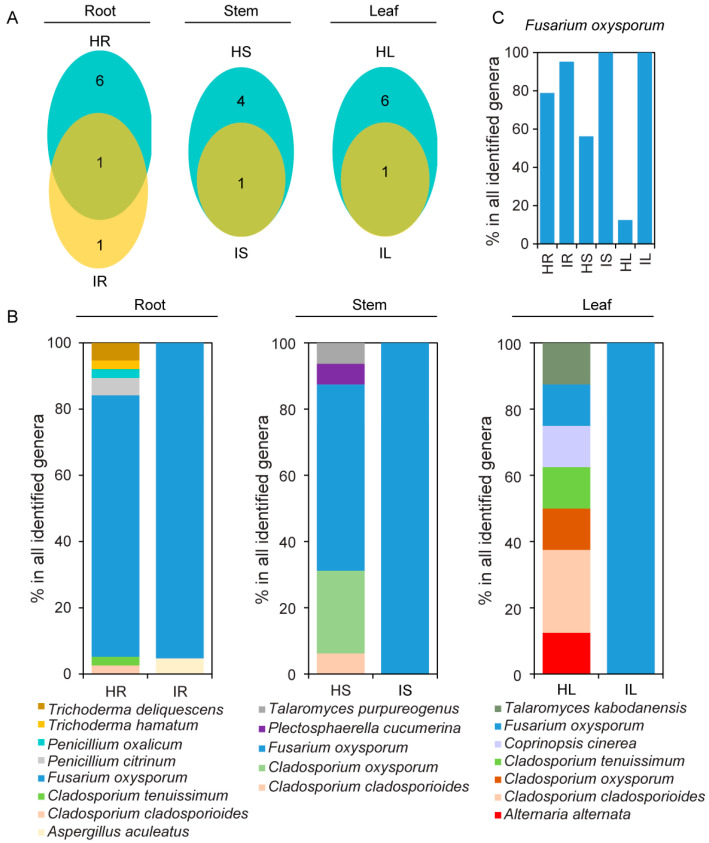
(**A**) Venn diagram of common and unique fungal species isolated using tissue separation from healthy and infected roots, stems, and leaves. (**B**) Relative abundance of fungal species *Fusarium oxysporum*. (**C**) Relative abundance of fungal species isolated from healthy and infected roots, stems, and leaves. HR: root samples of healthy strawberry plants; IR: root samples of infected strawberry plants; HS: stem samples of healthy strawberry plants; IS: stem samples of infected strawberry plants; HL: leaf samples of healthy strawberry plants; IL: leaf samples of infected strawberry plants.

Differences were observed between the results of the culture-dependent and culture-independent analyses used for determining the fungal community compositions of the healthy and infected samples (Figure 4B and Figure 6A–C). In the culture-dependent analysis, 62 fungal strains were isolated from the healthy strawberry samples, and 40 fungal strains were isolated from the infected strawberry samples, with *F. oxysporum* accounting for 64.52% and 97.50%, respectively, of these samples. Only one species (*F. oxysporum*) was identified in the roots, stems, and leaves of the healthy and infected strawberry samples, and six, four, and six unique fungal species were identified in the roots, stems, and leaves, respectively, of the healthy strawberry samples (Figure 6A,B).

According to our results, the proportion of beneficial fungal genera, such as *Trichoderma* spp., *Penicillium* spp., and *Talaromyces* spp., in the strawberries with *Fusarium* wilt was significantly lower, whereas the proportion of *F. oxysporum* was significantly higher, reaching 100% in the stems and leaves (Figure 6B,C). The proportion of *F. oxysporum* in the infected strawberry roots, stems, and leaves was significantly higher than that in the healthy strawberry roots, stems, and leaves, which is consistent with the trend of change in *Fusarium* identified through ITS high-throughput sequencing (Figure 4C and Figure 6C).

## 3. Discussion

Understanding the taxa and distribution of microbial communities in plants is crucial to the prevention and control of plant diseases [10,30,31]. Several species of *Fusarium*, particularly *F. oxysporum*, are well-known plant pathogens that result in wilting and economic losses in various plants [32,33,34]. In this study, our results indicated that, according to the Shannon and Chao1 indices, the diversity of the bacterial and fungal communities in the infected roots, stems, and leaves of the strawberry plants was lower than that in the healthy samples (Figure 1A–D), presumably because the plants’ vascular tissues were disrupted after infection with *Fusarium* wilt. This process led to the absolute dominance of *F. oxysporum*, with increased colonization, which altered the composition and distribution of the plants’ microbiome. These results are consistent with those of previous studies, which indicated that the diversity of bacterial and fungal communities in infected rhizosphere soil surrounding healthy strawberry plants was higher than that in powdery-mildew-infected samples [29]. Therefore, the high diversity of endophytic bacteria and fungi observed in healthy strawberry plants may play a key role in initiating host defenses against pathogen invasion, which may in turn increase the host’s resistance to pathogen invasion [35,36].

Overall, our results reveal a strong variation in the microbial taxonomic composition of the roots, stems, and leaves of healthy strawberry plants and *Fusarium* wilt strawberry plants. Alphaproteobacteria were more abundant in infected roots than in healthy roots, whereas Gammaproteobacteria were more abundant in infected stems and leaves than in healthy stems and leaves. The relative abundance of Firmicutes, Actinobacteria, and Chloroflexi was lower in infected rhizosphere roots, stems, and leaves than in healthy rhizosphere roots, stems, and leaves (Figure 3A). Multiple studies have indicated that Chloroflexi, Firmicutes, Actinobacteria, and Proteobacteria are associated with disease suppression [37,38,39]. In the current study, Sordariomycetes were more abundant in infected roots, stems, and leaves than in healthy samples, whereas Basidiomycota were less abundant in infected roots, stems, and leaves than in healthy samples (Figure 3B). These results are consistent with previous findings indicating that the relative abundance of Basidiomycota in the infected rhizosphere soil of healthy strawberry plants was higher than that in powdery-mildew-infected samples [29].

After infection with *Fusarium* wilt, the relative abundance of certain bacterial and fungal genera notably changed. For example, the relative abundance of *Pelomonas*, *Sphingobium*, *Ralstonia*, *Comamonas*, *Methylophilus*, *Lactobacillus*, *Streptomyces*, *Bacillus*, *Exiguobacterium*, *Aquabacterium*, and *Bradyrhizobium* decreased in the infected roots, stems, and leaves, whereas the relative abundance of *Pseudomonas* and *Microbacterium* exhibited the opposite trend (Figure 4A). These changes in the composition of different taxonomic groups were presumably due to *Fusarium* invasion. Generally, *Lactobacillus*, *Streptomyces*, *Bacillus*, *Pseudomonas*, and *Microbacterium* have the potential to prevent and control *Fusarium* pathogens [40,41,42,43,44,45,46]. These changes observed in the microbial composition of strawberry roots, stems, and leaves were presumably due to the plants’ resistance to *F. oxysporum* invasion and expansion, thus explaining their resistance to wilting. In terms of fungi, we detected *Fusarium* in all samples, with the proportions in healthy roots and infected roots, healthy stems and infected stems, and healthy leaves and infected leaves being 65.04% and 82.48%, 44.32% and 98.87%, and 8.94% and 53.94%, respectively (Figure 4B,C). These findings are consistent with the fact that *Fusarium* is the most commonly isolated genus in plant samples [47,48]. We also detected a higher abundance of *Fusarium* in the infected plant samples, which is consistent with this study’s field observations and culture isolation results for strawberry wilt (Figure 4C and Figure 6B,C). In the infected samples, the relative abundance of *Rhodotorula*, *Acremonium*, *Apiotrichum*, *Alternaria*, *Debaryomyces*, *Cadophora*, and *Sarocladium* exhibited a decreasing trend, which was presumably because *Fusarium* occupied a highly favorable ecological niche in the infected samples. The presence of endophytic *Fusarium* in the healthy samples may be a potential pathogenic fungus, which can be infected when the opportunity is appropriate, or it may not be pathogenic.

According to the PICRUSt results regarding bacterial metabolic function, most of the predicted protein sequences in the strawberry samples were clustered into metabolism (74.35%), environmental information processing (8.59%), cellular processes (5.85%), and genetic information processing (5.06%). In the metabolism cluster, global and overview maps, carbohydrate metabolism, and amino acid metabolism served as the primary pathways between healthy and infected strawberries. Compared with that observed in the roots, the bacterial function changes observed in the stems and leaves were more significant after infection with *Fusarium* wilt. The relative abundances of sequences related to amino acid metabolism and lipid metabolism were significantly lower in the infected stems and leaves than in the healthy stems and leaves (Figure 5A). A proteomic analysis revealed that the volatile organic compounds produced by biocontrol *Bacillus amyloliquefaciens* SQR-9 reduced the carbohydrate and amino acid metabolism of the protein of the tomato wilt pathogen *Ralstonia solanacearum* [49]. Moreover, the exogenous addition of mannitol and trehalose increased the production of chlamydospores of biocontrol fungal strains of *T. harzianum* T4, and enhanced their stress resistance by regulating lipid metabolism, indicating that lipid metabolism is an essential component of chlamydospore production and affects the stress resistance of chlamydospores [50]. These changes observed in the amino acid and lipid metabolism pathways may be related to the resistance of bacterial communities in strawberry stems and leaves to *Fusarium* infection.

In terms of the FUNGuild function prediction, a confidence level of “possible” or “highly probable” was selected to ensure prediction accuracy, whereas a confidence level of “possible”, such as *Fusarium*, was excluded. After these criteria were applied, the relative abundance of plant pathogens remained higher in the infected root, stem, and leaf samples than in the healthy root, stem, and leaf samples (Figure 5B). These results indicate that *Fusarium* infection may result in the accumulation of other plant pathogens in infected plants, presumably because of the destruction of the plant’s immune system due to *Fusarium* infection, which enables the entry of other pathogens to the plant tissues. For example, the co-inoculation of *Fusarium* and *Phytophthora sojae* into soybeans may increase the rate of infection with *P. sojae* [51].

After tissue separation, we discovered that the proportion of *F. oxysporum* in the infected strawberry roots, stems, and leaves was significantly higher than that in the healthy samples. We also discovered that the proportion of beneficial fungal genera, such as *Trichoderma* spp., *Penicillium* spp., and *Talaromyces* spp., in the strawberries with *Fusarium* wilt significantly decreased (Figure 6B,C). Previous studies have shown that several isolates of these fungal genera have been investigated as potential antagonistic agents against *Fusarium* pathogens [43,46,52]. These findings are consistent with that of the reduced diversity in the infected samples compared with that in the healthy samples, as detected using ITS high-throughput sequencing (Figure 1B). They are also consistent with previous research indicating that infection with plant pathogens may lead to changes in the endophytic community, thus resulting in a decrease in microbial diversity [53,54]. These changes may be due to the dominance of plant pathogens in plant tissues, whose presence may prevent the recruitment of beneficial microbes [9].

It should be mentioned that *F. oxysporum* is a fungal pathogen which produces high levels of mycotoxins, mainly including fusaric acid (FA), moniliformin, and fusarins [55,56,57,58]. These mycotoxins are suspected to be potent pathogenicity factor in plant disease development, including inhibiting plant growth and causing plant wilt [57,59]. Infected strawberry plants contained a large amount of *F. oxysporum*, which may produce high levels of mycotoxins that could have a strong impact on the fungal and bacterial communities of the strawberry plant.

## 4. Materials and Methods

### 4.1. Sample Collection

On 29 June 2021, strawberry samples were collected using random sampling during the seedling stage from a strawberry greenhouse (temperature: 26–28 °C, humidity: 60–80%, and plant spacing: 50 cm) located in Jiangsu Agricultural Expo Park, Jiangsu Province, China (32°02′ N, 119°26′ E). These strawberry samples belonged to the cultivar Beni Hoppe, which had been continuously planted for 2 years. A total of 60 samples were collected, including 10 root samples of healthy strawberries (HR), 10 root samples of wilt-infected strawberries (IR), 10 stem samples of healthy strawberries (HS), 10 stem samples of wilt-infected strawberries (IS), 10 leaf samples of healthy strawberries (HL), and 10 leaf samples of wilt-infected strawberries (IL). Each sample was separately collected, placed in a sterile plastic bag, and transported on ice to a laboratory.

### 4.2. Sample Preparation and DNA Extraction

Briefly, all strawberries were rinsed with running water to remove soil residue and dust. Subsequently, the roots, stems, and leaves of each strawberry plant were cut off and evenly mixed, and 2 g of each sample was randomly weighed for surface disinfection. To ensure the removal of all epiphytic microbes, 100 mL of sterile water and two drops of Tween 20 were added, and the mixture was shaken at 220 rpm at 25 °C for 20 min; treated with sterile water for 20 s, 70% (*v*/*v*) ethanol for 30 s, and 2.5% (*v*/*v*) sodium hypochlorite solution for 2 min; and finally rinsed with sterile water three or four times. Each part of the root, stem, and leaf was further cut into shorter segments (0.25 cm), and each sample was divided into two portions for culture-dependent and culture-independent analysis, as described in the following:

In the culture-dependent analysis of fungi, five segments of each sample were randomly selected and placed on a plate containing potato dextrose agar (PDA) with three replicates and were cultured at 25 °C. Ampicillin (50 mg/L) and rifampicin (50 mg/L) were added to all media in advance to inhibit the growth of bacteria. After 3–5 days, when a mycelium emerged from the tissue block, a small piece of medium growing on the edge of the medium was carefully transferred together with the mycelium to a new plate containing PDA. On the basis of the type of mycelia, colony color, and growth rate, these pure fungal cultures were preliminarily divided into morphological taxa. After 7 days of growth, mycelial DNA was extracted using a DNAsecure Plant Kit (Tiangen, Beijing, China) as per the manufacturer’s instructions. Subsequently, PCR amplification was performed using the primers ITS1 and ITS4 in accordance with previously outlined protocols [60] (Table S2). We amplified the translation elongation factor 1-α (EF1-α/TEF1) and β-tubulin (BenA) to confirm species of the *Fusarium* [61], *Trichoderma* [62], and *Talaromyces*, respectively [63] (Table S2). The PCR reaction system and amplification program were carried out according to the established protocols [60]. After the PCR products were sequenced, their sequences were compared using the NCBI database (BLASTN, http://www.ncbi.nlm.nih.gov (accessed on 25 February 2022)) for species identification.

In the culture-independent analysis method, each remaining sample was placed in a sterilization mortar, soaked with liquid nitrogen, and ground with a pestle. DNA was subsequently extracted using the aforementioned kit in accordance with the manufacturer’s instructions. The DNA concentration and purity were quantified using a NanoDrop 2000 spectrophotometer (Thermo Fisher Scientific, Wilmington, DE, USA).

### 4.3. PCR Amplification and Illumina MiSeq Sequencing

Bacterial 16S rRNA gene fragments (V5–V7) were amplified using the primers 799F (5′-AACMGGATTAGATACCCKG-3′) and 1193R (5′-ACGTCATCCCCACCTTCC-3′) from the extracted DNA [64,65]. PCR was performed using 4 μL of 5× FastPfu buffer, 2 μL of 2.5 mM deoxynucleotide triphosphates (dNTPs), 0.8 μL of each primer (5 μM), 0.4 μL of TransStart FastPfu DNA Polymerase, 0.2 μL of bovine serum albumin (BSA), and 10 ng of template DNA, and ddH_2_O was added until the mixture reached a final volume of 20 μL. Amplification was performed as follows: an initial round at 95 °C for 3 min followed by 27 cycles at 95 °C for 30 s, 55 °C for 30 s, and 72 °C for 45 s; a second round comprising 13 cycles at 95 °C for 30 s, 55 °C for 30 s, and 72 °C for 45 s; and a final extension round starting at 72 °C for 10 min and ending at 4 °C. The fungal ITS1 region was amplified using the primers ITS1F (5′-CTTGGTCATTTAGAGGAAGTAA-3′) and ITS2 (5′-GCTGCGTTCTTCATCGATGC-3′) [32,66,67]. PCR was performed using 2 μL of 10×buffer, 2 μL of 2.5 mM dNTPs, 0.8 μL of each primer (5 μM), 0.2 μL of TaKaRa rTaq DNA Polymerase, 0.2 μL of BSA, and 10 ng of template DNA, and ddH_2_O was added until the mixture reached a final volume of 20 μL. Amplification was performed as follows: an initial round at 95 °C for 3 min, followed by 36 cycles at 95 °C for 30 s, 53 °C for 30 s, and 72 °C for 45 s, and a final extension round starting at 72 °C for 10 min and ending at 4 °C. The PCR amplification results were detected using 2% agarose gel electrophoresis. Amplicons were subjected to paired-end sequencing on an Illumina MiSeq sequencing platform with a PE300 (Shanghai Majorbio Bio-Pharm Technology, Shanghai, China).

### 4.4. Amplicon Sequence Processing and Analysis

After the demultiplexing was completed, the resulting sequences were merged using FLASH v.1.2.11 [68] and quality-filtered using fastp v.0.19.6 [69]. Operational taxonomic units (OTUs) with a similarity cutoff of 97% were clustered using UPARSE v.11 [70], and chimeric sequences were identified and removed. The taxonomy of each OTU representative sequence of bacteria and fungi was analyzed using RDP Classifier v.2.13 [71] against the SILVA database v.138 [72] and UNITE database v.8.0 [73], with a confidence threshold of 0.7.

We estimated the fungal and bacterial community diversity (Shannon’s and Simpson’s indices) and community richness (Chao1, Ace, and Sobs indices) in the healthy and wilt-infected strawberry roots, stems, and leaves using mothur v.1.30.2 (http://www.mothur.org/wiki/Calculators (accessed on 25 February 2022)) [74]. A principal coordinates analysis (PCoA) of the Bray–Curtis distances was performed with the vegan package in R v.3.3.1. ANOSIMs based on the Bray–Curtis distances were performed to evaluate the significant differences between healthy and wilt-infected strawberries and compartments via the vegan package of R v.3.3.1.

OTUs classified as “Cyanobacteria” or “Mitochondria” were excluded from the OTU table of bacteria, and OTUs classified as “norank” or “unclassified_k__Fungi” were excluded from the OTU table of fungi. The potential functions of the bacterial and fungal communities in the healthy and wilt-infected strawberry roots, stems, and leaves were estimated using PICRUSt2 (http://www.genome.jp/kegg/ (accessed on 25 February 2022)) [75,76] and FUNGuild (http://www.stbates.org/guilds/app.php (accessed on 25 February 2022)) [77,78] calculations, respectively.

### 4.5. Statistical Analysis

Student’s *t* test was used to compare the alpha diversity indices, the relative abundance of bacterial and fungal taxa (phylum and genus), and the relative abundance of bacterial metabolic function and fungal functional guilds in the healthy and infected roots, stems, and leaves (*p* < 0.05). All statistical analyses were conducted using IBM SPSS Statistics v.20.0 (IBM, Armonk, NY, USA).

## 5. Conclusions

In this study, we investigated the changes that occur in the microbial communities of strawberry plants after infection with *Fusarium*. Our results reveal significant variations in the bacterial and fungal communities in the roots, stems, and leaves of healthy and infected samples. After infection with *Fusarium*, *F. oxysporum* rapidly occupied the ecological niche of the strawberry plants, resulting in significant changes in the composition of the microbial communities in the roots, stems, and leaves. The diversity of the bacterial and fungal communities decreased, and the number of beneficial microorganisms within the plants also decreased, thereby enabling other plant pathogens to enter the plants. Overall, our findings can serve as a theoretical basis and biocontrol resource for the prevention and control of *Fusarium* wilt in strawberry plants.

## Data Availability

Raw sequence reads are available in the NCBI Sequence Read Archive (SRA) database under the accession number PRJNA1036132.

## Supplementary Materials

The following supporting information can be downloaded at https://www.mdpi.com/article/10.3390/plants12244153/s1: Figure S1: PCoA based on Bray–Curtis differential analysis indicating effect of *Fusarium* infection on composition of bacterial (A) and fungal (B) communities in strawberry roots, stems, and leaves. ANOSIM conducted to test for differences in community composition resulting from *Fusarium* infection. *R* values labeled with asterisks: * *p* < 0.05; Table S1: Alpha diversity of bacteria and fungi in healthy and infected strawberry roots, stems, and leaves. Values presented as means ± standard deviations for healthy and infected strawberry roots, stems, and leaves (*n* = 10). Uppercase letters representing *p* < 0.01 level and lowercase letters representing *p* < 0.05 level, different letters represent significant differences according to Student’s *t* test; Table S2: The primers used in this study.
