# Supplementary material for: Fusarium Wilt Invasion Results in a Strong Impact on Strawberry Microbiomes
Source: Plants (Basel). 2023 Dec 13;12(24):4153. doi: 10.3390/plants12244153 (PMC10747085; doi:10.3390/plants12244153)
Supplement: Supplementary file 1 [file plants-12-04153-s001.zip › Figure S1.pdf]

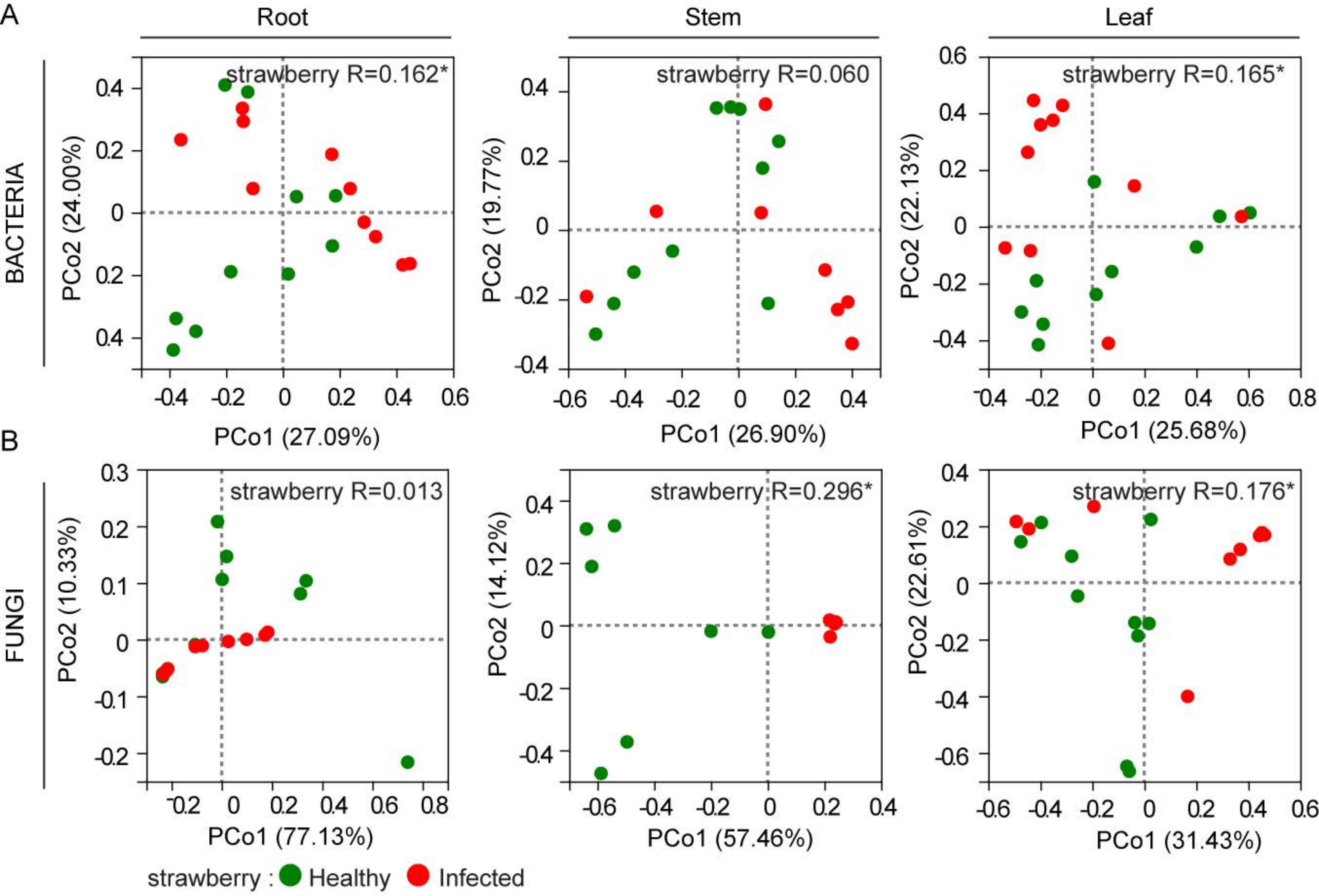

Figure S1. PCoA based on Bray–Curtis differential analysis indicating effect of *Fusarium* infection on composition of bacterial (**A**) and fungal (**B**) communities in strawberry roots, stems, and leaves. ANOSIM conducted to test for differences in community composition resulting from *Fusarium* infection. *R* values labeled with asterisks: * $p < 0.05$.
